# Computationally Designed Peptides for Zika Virus Detection: An Incremental Construction Approach

**DOI:** 10.3390/biom9090498

**Published:** 2019-09-17

**Authors:** Marcello Mascini, Emre Dikici, Marta Robles Mañueco, Julio A. Perez-Erviti, Sapna K. Deo, Dario Compagnone, Joseph Wang, José M. Pingarrón, Sylvia Daunert

**Affiliations:** 1 Department of Analytical Chemistry, Faculty of Chemistry, University Complutense of Madrid, Ciudad Universitaria s/n, 28040 Madrid, Spain; pingarro@ucm.es; 2 Faculty of Bioscience and Technology for Food, Agriculture and Environment, University of Teramo, 64100 Teramo, Italy; dcompagnone@unite.it; 3 Department of Biochemistry and Molecular Biology, Miller School of Medicine, University of Miami, Miami, FL 33136, USA; edikici@med.miami.edu (E.D.); mroblesma@gmail.com (M.R.M.); sdeo@med.miami.edu (S.K.D.); 4 Dr. JT Macdonald Foundation Biomedical Nanotechnology Institute, University of Miami, Miami, FL 33136, USA; 5 Center for Protein Studies, Faculty of Biology, University of Havana, Havana 10400, Cuba; japerviti@gmail.com; 6 Department of Nanoengineering, University of California, San Diego, La Jolla, CA 92093, USA; josephwang@eng.ucsd.edu; 7 University of Miami Clinical and Translational Science Institute, University of Miami, Miami, FL 33136, USA

**Keywords:** virtual screening, molecular modelling, peptides, Zika virus, Dengue virus, antibody mimetics, ELISA

## Abstract

Herein, and in contrast to current production of anti-Zika virus antibodies, we propose a semi-combinatorial virtual strategy to select short peptides as biomimetic antibodies/binding agents for the detection of intact Zika virus (ZIKV) particles. The virtual approach was based on generating different docking cycles of tetra, penta, hexa, and heptapeptide libraries by maximizing the discrimination between the amino acid motif in the ZIKV and dengue virus (DENV) envelope protein glycosylation site. Eight peptides, two for each length (tetra, penta, hexa, and heptapeptide) were then synthesized and tested vs. intact ZIKV particles by using a direct enzyme linked immunosorbent assay (ELISA). As a reference, we employed a well-established anti-ZIKV antibody, the antibody 4G2. Three peptide-based assays had good detection limits with dynamic range starting from 10^5^ copies/mL of intact ZIKV particles; this was one order magnitude lower than the other peptides or antibodies. These three peptides showed slight cross-reactivity against the three serotypes of DENV (DENV-1, -2, and -3) at a concentration of 10^6^ copies/mL of intact virus particles, but the discrimination between the DENV and ZIKV was lost when the coating concentration was increased to 10^7^ copies/mL of the virus. The sensitivity of the peptides was tested in the presence of two biological matrices, serum and urine diluted 1:10 and 1:1, respectively. The detection limits decreased about one order of magnitude for ZIKV detection in serum or urine, albeit still having for two of the three peptides tested a distinct analytical signal starting from 10^6^ copies/mL, the concentration of ZIKV in acute infection.

## 1. Introduction

The threat of Zika infection has emerged as a global public health problem because of its ability to cause severe congenital disease and affect a large population [1,2]. Zika infection is known to cause neurological problems to pregnant women and potentially cause microcephaly and other congenital malformations and diseases to the unborn child. Zika affects both males and females, and it has been reported that the virus can be transmitted sexually through semen and vaginal fluids. The Zika virus (ZIKV) is a mosquito-borne flavivirus that shares many common genetic sequences and protein structures with other flaviviruses. Because of the lack of specific antibodies/binders that can be used for the diagnosis of the disease, the current bioassays present cross-reactivity with other flaviviruses and arboviruses, especially with the Dengue virus (DENV) [3,4].

This limits the use of immunoassays for the detection of human pathogens within the flavivirus genus [5,6]. Thus, there is a need for highly selective binders for ZIKV that can be employed in diagnostics and health status assessment of patients suffering from Zika.

The ZIKV, like other flavivirus, is composed of three structural proteins (capsid, pre-membrane, and envelope) and seven nonstructural proteins. The flavivirus envelope protein mediates viral assembly, attachment, entry, and fusion, and constitutes a major target for neutralizing antibodies. The ZIKV envelope protein is divided into three domains: a central β-barrel domain (DI), an extended dimerization domain (DII), and an immunoglobulin-like segment (DIII) [7].

The ZIKV envelope protein structure is similar to other known flaviviruses envelope protein except for the ~10 amino acids that surround the Asn 154 glycosylation site found in each of the 180 envelope glycoproteins that makeup the icosahedral shell [8].

In this work, the ZIKV glycosylation site was taken as the target for designing selective peptides for ZIKV by means of a semi combinatorial virtual strategy. The virtual approach was based on generating different docking cycles of tetra, penta, hexa, and heptapeptide libraries by maximizing the discrimination between the amino acid motif in the ZIKV and DENV glycosylation binding site. DENV was chosen among the flavivirus family because it presents structural similarities with ZIKV. Moreover, Zika and Dengue have similar geographical distribution and initial symptoms but different potential complications. Zika is associated with microcephaly in newborns [9], whereas dengue can result in severe life-threatening complications like dengue hemorrhagic fever or dengue shock syndrome [10]. No specific therapeutic options exist for ZIKV or DENV infections, and successful case management, surveillance, and control depends on correct identification and discrimination between both the viral infections [11].

Peptides as antibody mimetic elements in diagnostic methods were recently reviewed highlighting the features desired to outperform the antibodies with regard to binding affinities, cellular and tumor penetration, large-scale production, temperature, and pH stability [12]. It is well documented that a harsh chemical environment can affect the antibodies binding properties, and DNA and peptides aptamers are the most promising candidates to replace them in bioanalysis as reported by recent reviews [13,14,15]. Aptamers have become increasingly important molecular tools for diagnostics and as therapeutic agents, and are used in many analytical applications, such as chromatography, electrophoresis, mass spectrometry, molecular beacons, gas sensors, and biosensors [16,17,18,19,20].

In recent works, short peptides were used as molecular binders for virus detection. Linear peptides were selected by phage display to detect norovirus using the enzyme linked immunosorbent assay (ELISA) protocol or by means of an impedance biosensor [21,22].

Computationally designed peptides were used to detect flavivirus. Binding affinity and stability of disulfide cyclic peptide ligands targeting DENV envelope glycoprotein were calculated by molecular docking and molecular dynamics simulation, but no experimental evidence was provided [23].

A recent report predicted the structure of short peptides targeting the ZIKV envelope protein by molecular modelling and docking simulation [24]. Do Thi Hoang Kim et al. (2018) tested the interactions between the selected peptides and virus via a fluorescence-linked sandwich immunosorbent assay (FLISA), and the performance of the peptide-linked sandwich FLISA was evaluated in virus-spiked human serum and urine.

Molecular modelling is more and more used to overcome the trial and error approach and to minimize the experimental problems by providing an understanding of atomic interactions and facilitating the rational design of experimental protocols [25,26,27,28,29]. Virtual docking is currently an important tool in drug discovery, and a subject of important developments over the last decade [30,31].

However, a number of obstacles still limit the widespread use of molecular modelling for biotechnological applications. One of the most important drawbacks for mainstream use of molecular modeling is the challenge to simulate a huge number of candidates to be designed or/and docked using a full combinatorial approach.

To address this issue, herein we present a new methodology, based on an incremental construction approach to choose short peptides as binding agents for the selective detection of the intact ZIKV particles. Synthetic peptides are more resistant to physicochemical stress, more reproducible and less expensive when compared with antibodies, so even if they show less specificity, they can be used as an array giving synergetic contribution to the detection.

Direct ELISA was chosen as the experimental protocol to check the performances of the peptides. ELISA was preferred to other analytical techniques because it provides automated steps to speed-up the screening of a large number of experimental trials.

## 2. Materials and Methods

### 2.1. Virtual Docking

All calculations of molecular docking were done using a desktop computer with 19 processors Intel Xeon X5690 at 3.47 GHz each, with 94.5 GiB RAM, running Kernel Linux 2.6.32-642.1.1el6.x86_64, GNOME 2.28.2. The four peptide libraries were designed and cleaned up with HyperChem 8.0.5.

Peptides were designed in zwitterionic mode, using only the 20 natural amino acids, adding hydrogens at pH 7, using molecular mechanics method amber, with the “Steepest Descent” algorithm converging at 0.08 kJ mol^−1^ in 32767 as maximum of cycles. The secondary structure setting was left in HyperChem default mode (beta-sheet with Psi and Phi set to 180° and L-isomer). The script running in HyperChem automatically removed peptide duplicates. Minimization, conformer generation, and docking were carried out using OpenEye Scientific Software package under academic license. Each peptide library was compacted in a single file and fast minimized in vacuum to reduce computing time. In this context, solvent condition did not change significantly the results. The energy minimization process was carried out using SZYBKI 1.5.7 in its default parameterization [32]. To take into account the flexibility of the peptides, ten conformers were generated for each peptide by means of the OMEGA 2.4.6 used with MMFF as the force field [33,34,35]. Therefore, the ligands were represented by the peptide conformers, around 5 million units.

Then the envelope proteins, taken as the receptors, were downloaded from the protein data bank website. The envelope proteins were from the flavivirus species ZIKV and DENV-2, having respectively the following codes in the Protein Data Bank web site: 5IRE [7] and 4UTC [36]. All residues and water molecules were removed from the envelope proteins pdb files. For each envelope protein, a dedicated box enclosing the glycosylation site was generated. The active site box, defining the general space of the protein where peptides are expected to bind, was designed around the amino acid Asn 154 in 5IRE and the Asn 153 in 4UTC. In order to reduce the calculation time, tetra and pentapeptide libraries were docked using active site boxes with a volume of around 13 nm^3^ and hexa and heptapeptide libraries using boxes having a volume of around 18 nm^3^.

Using these sizes, the entire molecular surface of all peptide conformers was inside the active site box.

The active site box along with the multi-conformer rigid body docking were carried out using OEDocking 3.0.0 [37,38]. Multi-conformer rigid body docking was run using Chemgauss4 as scoring function. The Chemgauss4, a modification of the Chemgauss3, was the latest scoring function from OpenEye software with improved hydrogen bonding and metal chelator functions. The total score obtained was the sum of steric, acceptor/metal, donor, and aromatic contributions. The rigid docking approach was used with the purpose of saving machine time, allowing complex calculations to be run using modest resources within a reasonable timeframe.

Structures visualization and generation of molecular surfaces were performed using VIDA 4.1.1 [39] and PyMOL [40].

The sequence identity between ZIKV and DENV strain 2 (DENV-2) envelope protein was calculated performing a multiple sequence alignment with the program Clustal Omega [41] using the default parameters. The protein sequences used as input were extracted from Uniprot (www.uniprot.org) (ZIKV accession code: A0A024B7W1, residues 291-794; DENV-2 accession code: P07564, residues 291-794).

The entire process was automated using a bash script and using a freeware BASIC-like scripting language (AutoIT V3) for post processing data analysis.

Physico-chemical properties were calculated using VIDA 4.1.1 and a peptide property calculator web site (www.pepcalc.com/).

### 2.2. Experimental Setup

All chemicals used for buffers were of analytical grade and purchased from Sigma-Aldrich available online at: http://www.sigmaaldrich.com (accessed on 1 August 2019).

The eight peptides were purchased from Biomatik available online at: http://www.biomatik.com (accessed on 15 July 2019). A cysteine was added to all peptides to bind maleimide-PEG_2_-biotin, used to label each of the peptides with the signal amplifier streptavidin-HRP. All peptides were provided with a purity >85%.

Lyophilized peptides were diluted at 1 mM concentration in 10 mM phosphate buffered saline (PBS) pH 7.4, divided into 100 μL aliquots and stored at −30° C for further use.

Before biotin functionalization, peptides stock solution was reduced using Tris[2-carboxyethyl] phosphine hydrochloride (TCEP) disulfide reducing gel from ThermoFisher Scientific available online at: www.thermofisher.com (accessed on 15 April 2019) and after 1 h, the gel was removed using TCEP gel spin separation columns (ThermoFisher Scientific). Then, two-fold molar excess of EZ-Link™ Maleimide-PEG2-Biotin (ThermoFisher Scientific) was added to purified peptide solution and incubated for 1 h. At this concentration, EZ-Link™ Maleimide-PEG2-Biotin did not contribute to background signal as shown by a pilot test using only EZ-Link™ Maleimide-PEG2-Biotin without peptide. Therefore, no further separation was carried out.

To optimize all parameters of the direct ELISA protocol, Pierce 96-well polystyrene plates, (ThermoFisher Scientific) were coated overnight at 4° C with different concentrations of intact virus particles (ZIKV or DENV). The intact virus particles were diluted using 100 mM NaHCO_3_, pH 9.6, and aliquots of 100 μL were dispensed into each well of the plate using a multichannel pipette. This buffer pH assured a strong hydrophobic binding interaction between polystyrene and virus particles.

Intact particles both ZIKV and DENV were provided by Dr. Watkins group (University of Miami, Dep. of Pathology). The samples were controlled and counted by focus forming assay and RT-PCR, the details were reported in a previous work [42]. The intact particles of ZIKV were inactivated using gamma irradiation. Assay biohazardous steps were carried out according to standard safety procedures.

The antibody 4G2 hybridoma mouse IgG2a was used as the reference and employed in combination with an anti-mouse IgG conjugated to HRP.

After coating the plates overnight, the intact virus particles were removed by washing five times with the washing buffer (PBST) 10 mM PBS pH 7.4, 0.1% Tween-20, using an automated plate washer (MultiWash+, Molecular Devices, Sunnyvale, CA). Then, the plates were blocked with 200 μL of blocking buffers while shaking at 300 rpm at room temperature. The blocking buffers used were Pierce™ protein-free (PBS) blocking buffer (PF), Blocker™ BLOTTO (BT) in TBS, SuperBlock^TM^ blocking buffer, Blocker™ BSA (1X) in PBS all from ThermoFisher Scientific. After 2 h, the blocking buffers were removed using the same washing procedure mentioned above. Hundred microliter aliquots of several dilutions of peptides biotinylated in 10 mM PBS pH 7.4 were placed in each well and incubated for 2 h while shaking at 300 rpm at room temperature. After the incubation, the unreacted peptides were removed by using the plate washer with the same settings. Then, 100 μL-aliquots of streptavidin-HRP (ThermoFisher Scientific) at a concentration of 20 ng/mL were added into each well and incubated for 30 min at room temperature without shaking. After the incubation, excess streptavidin-HRP was removed and the wells were washed with the plate washer five times using the washing buffer. Finally, 100 μL-aliquots of the Ultra TMB-ELISA substrate solution (ThermoFisher Scientific) were added and after 10 min, the reaction was stopped by adding 100 μL aliquots of the TMB stop solution from SeraCare, available online at; https://www.seracare.com (accessed on 1 abril 2019). The emission (450 nm) was read using a microplate reader (Clariostar Optima; BMG Labtech, Ortenberg, Germany).

The corresponding blank signals in triplicates were obtained by using all reagents without peptides. They were subtracted to the average absorbance values for triplicate wells of each test.

The statistical analysis, the data processing, and data fitting were carried out using the excel Office 365 add-ins XLSTAT available on line at: www.xlstat.com (accessed on 1 August 2019). Data (n = 3) were shown as average (Av) and standard deviation (SD). Statistical significance between ZIKV and DENV serotypes (strain 1, 2 and 3) was calculated using two-way analysis of variance.

## 3. Results and Discussion

### 3.1. Docking Simulations

According to multiple sequence alignment, ZIKV and DENV envelope protein were homologous with 52% sequence identity at amino acid level. However, the sequence identity is only 23% when the alignment is restricted to 24 amino acids around the asparagine residue in the glycosylation site, suggesting that this site is a poor conserved region between both proteins.

**Figure 1:**
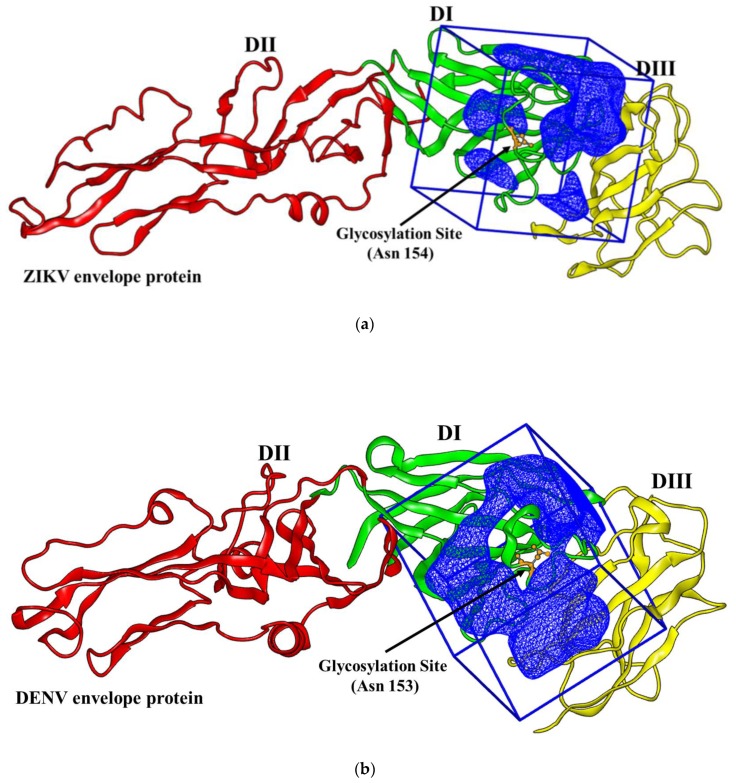
(**a**) 3D representation of the Zika virus (ZIKV) envelope protein (PDB code: 5IRE) (**b**) 3D representation of the dengue virus (DENV) envelope protein. (PDB code: 4UTC). Protein domains DI, DII, and DIII are depicted in green, red, and yellow, respectively. The glycosylation site residue (Asn 154 in ZIKV and Asn 153 in DENV) is displayed in orange. The blue meshes represent the outer docking contour of the envelope protein binding site inside the box chosen for docking the peptide libraries.

Given the above-mentioned distinction between the ZIKV and DENV structure, the ZIKV and DENV glycosylation sites were used as the receptor docking site to study the virtual binding interaction of the peptide libraries taken as the ligands. Figure 1 depicts the ZIKV and DENV envelope protein glycosylation site (PDB code: 5IRE and 4UTC). The outer docking contour inside the box, representing the volume of the protein binding site chosen for docking the peptide libraries, was highlighted in blue.

**Figure 2:**
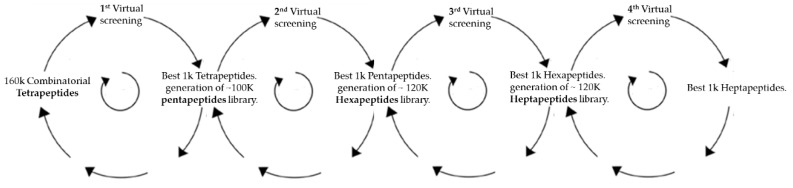
Schematic representation of the incremental construction approach for obtaining penta, hexa, and heptapeptides libraries. The semi-combinatorial approach was based on generating different cycles of libraries by maximizing the discrimination between amino acid motif in the ZIKV and DENV binding site. The best 1K peptides of every virtual screen were defined as the peptide ligands that were inside the top 5% peptides binding the ZIKV active site that ranked outside the top 5% when binding the DENV active site.

To our knowledge, only one other work used molecular modelling simulation to choose short peptides selective for ZIKV [24]. Do Thi Hoang Kim et al. (2018) designed ZIKV-specific peptide candidates using epitope prediction molecular modelling tools and then the top scoring peptides were modified to increase sensitivity to the ZIKV envelope protein and decrease sensitivity to the DENV envelope protein. The present work focused on the ZIKV glycosylation binding site having the greatest amino acidic motif difference within flavivirus family and used a semi-combinatorial virtual strategy to select peptides. The semi-combinatorial virtual strategy was run in four steps. (Figure 2) In each step, a peptide ligand library was generated by using an incremental construction approach. In every subsequent iteration, a focused library of peptides of increasing complexity was built on previous iteration results. The first peptide library docked was made by the entire 160K possible tetrapeptide combinations of the 20 natural amino acids.

The docking program used in this work was based on multi-conformer rigid body docking. In a previous work we studied the number of conformers to span the conformation space of small peptides and ten conformers were found to be a good option to represent the peptide conformational space with fair speed-accuracy ratio [43]. The overall binding tendency remained unchanged when using more than ten conformers, therefore ten conformers per peptide were generated to ensure a good compromise between the calculation time and accuracy of the output data for this type of ligands.

From the 5% peptide ligands (8k elements in tetrapeptides library) having the best biding score for ZIKV binding site, only 1K tetrapeptides were selected for the next step.

**Figure 3:**
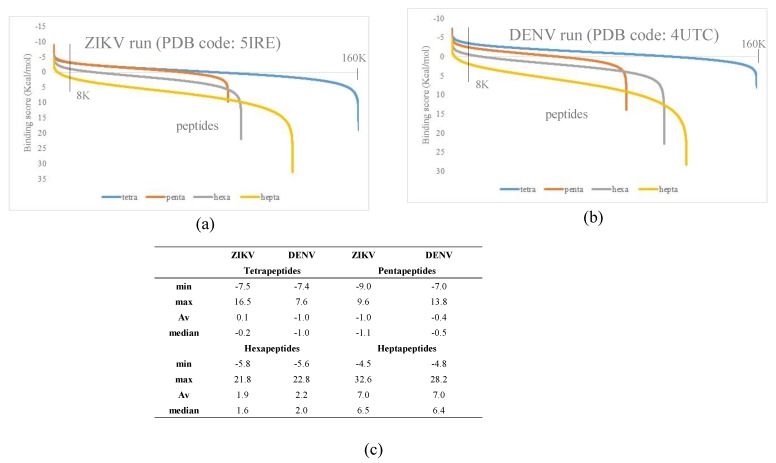
(**a**) Binding score trend of the four peptides libraries docked in ZIKV active site. (**b**) Binding score trend of the four peptides libraries docked in DENV active site. The graphs show the typical scores distribution obtained in the simulations. The data are sorted in ascending order of score, thus not necessarily a correspondence must exist between the positions of the peptides in each curve. (**c**) Statistical parameters of the score behavior (Kcal/mol), obtained using the four peptide libraries docked in the ZIKV and DENV envelope protein glycosylation binding site.

The criterion of the selection was to choose the peptide ligands inside the top 5% peptides binding the ZIKV active site that also ranked outside the top 5% when binding the DENV active site. The meaning of this selection was to maximize the discrimination between the amino acid motif in the ZIKV and DENV binding site. The 5% was selected as cut-off because in all simulations, this value delimited the segment of the curve in which the steeper slope change was observed (Figure 3a,b).

This criterion was also applied to the other steps to select penta, hexa, and heptapeptides. Figure 3a,b depicts the typical distributions of scores obtained in the simulations. Lower score values represent higher protein–peptide affinity. The curves obtained had similar Gaussian distributions. All docking runs had approximately 5% of the complexes with higher scores and 5% with worse scores, both well separated from the rest of the population (Figure 3a,b).

The second step was the generation of the pentapeptide library by using as scaffold the 1K tetrapeptides selected in the previous step. In each of the 1K tetrapeptides, all the 20 natural amino acids were inserted one by one before the first amino acid position while all four other positions were held fixed. Then, the same procedure was carried out between the first and second position and then between second and third position and so on. Using this approach, it was possible to select a minimum fraction (100K pentapeptides) of the entire pentapeptides sample space, which would result in 3.2 million combinations if freely varying the residues at all positions.

As reported in Figure 2, the hexa and heptapeptide libraries were built by using the same semi-combinatorial approach carried out in the second step but selecting for the hexapeptide library (third step) the best 1K pentapeptides and for the heptapeptide library (fourth step) the best 1K hexapeptides. A total of around 520K peptide ligands were docked using as receptor the glycosylation site of ZIKV and DENV. Figure 3c reports the statistical summary of the binding scores calculated for the four libraries of peptides toward the two envelope proteins. Score values comprised within the range from −9 to 33 Kcal/mol in all simulations. All peptide libraries showed average and median values very close to each other, demonstrating a good symmetry in normal distribution. As showed by the data, the peptide size played a critical role in both envelope proteins binding site interaction. The predicted binding drastically weakened as the ligands length increased from pentapeptides to heptapeptides. The score values were calculated using chemgauss4 scoring function and, thus, lower values meant higher protein–peptide affinity.

**Figure 4:**
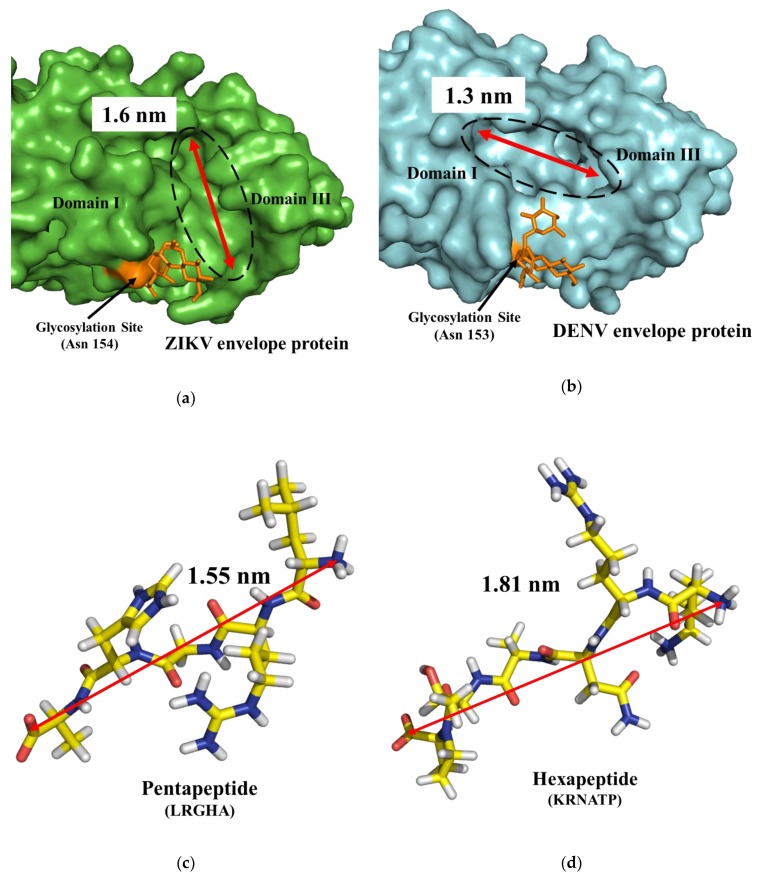
(**a**) 3D surface view of the ZIKV envelope protein (green). (**b**) 3D surface view of the DENV envelope protein (cyan). The glycosylation site is highlighted in orange. The pocket in both binding sites is highlighted by a black ellipse. The red arrow illustrates the length of pocket. (**c**) 3D representation of pentapeptide LRGHA. (**d**) 3D representation of hexapeptide KRNATP. The red arrow illustrates the peptide length, measured from N-terminus to C-terminus.

These results could be explained considering the steric effects of the ligands within the binding pockets of the envelope proteins. As shown by Figure 4a, the ZIKV envelope protein presents a 1.6 nm long pocket near the glycosylation site that acts as a binding site for most docked peptides. The ellipse major axis length of this cavity is similar to pentapeptides length (1.5–1.6 nm), but shorter than hexapeptides (1.8–1.9 nm) (Figure 4c,d). Tetrapeptides and pentapeptides can easily bind within the pocket thus presenting higher binding score than hexapeptides and heptapeptides that are conformationally hindered. In addition, the minimum–maximum range among the peptide libraries reflected that this behavior becomes relevant for hexapeptides, and even more for heptapeptides. The steric clashes can justify also the differences with the docking results in the DENV binding site, which has a 1.3 nm long pocket in a similar position but with slightly different amino acid composition (Figure 4b).

The statistical analysis of the residues surrounding the ZIKV and DENV glycosylation site highlighted slightly the differences in amino acid composition between both receptor binding sites. (Table 1a and Table S1 in Supplementary Materials). In both receptor sites, aliphatic residues had the highest occurrence (45% in ZIKV and 49% in DENV), followed by polar residues (26% and 19%, respectively), positively charged residues (13% and 15%), negatively charged residues (12% and 13%) and aromatic residues (4% in both receptors). Nevertheless, the ZIKV binding site displayed less aliphatic and charged residues but more polar residues than DENV site, suggesting differences in the glycosylation binding site that can be used for discriminating between both viral proteins.

Table 1b reports the data of the statistical analysis carried out on the 0.1% best ranked peptides vs. both ZIKV and DENV. This fraction was selected because in all peptide libraries the simulated binding energy decreased exponentially in the top 0.1% best ranked peptides. A decrease of almost 30% in the binding score was observed from 1st to ~100th peptide position in all peptide libraries. Aliphatic residues had the highest occurrence among the docked peptides, comprising around 50% of their average amino acid composition. Remarkably, in most of DENV top peptides the incidence of aliphatic residues was higher compared to ZIKV top peptides (44% vs. 61% in tetrapeptides, 51% vs. 60% in pentapeptides and 49% vs. 60% in heptapeptides), with the notable exception of hexapeptides, where the residue occurrence was similar (50% vs. 46%). Polar residues were the second most common amino acids found in docked peptides, with similar occurrence in both ZIKV and DENV docked pentapeptides (32% in both cases) and heptapeptides (36% vs. 32%), but differences in tetrapeptides (31% vs. 19%) and hexapeptides (33% vs. 42%). Positively charged residues were far less common in the peptide composition, with its highest occurrence in tetrapeptides (14% in both ZIKV and DENV) and lowest in heptapeptides (9% in ZIKV and 3% in DENV). Overall, these residues were more common in ZIKV docked peptides than DENV (9% vs. 6%, in pentapeptides, 10% vs. 3% in hexapeptides, and 9% vs. 3% in heptapeptides), with the exception of tetrapeptides, where the occurrence was similar in both ZIKV and DENV (14% in both cases). Aromatic residues were poor represented among top peptides, with higher occurrence in ZIKV tetra and pentapeptides in comparison with DENV ones (8% vs. 5% in tetrapeptides, 7% vs. 2% in pentapeptides). However, this behavior was not observed in hexapeptides (5% ZIKV vs. 8% DENV), and heptapeptides (4% in both ZIKV and DENV). Negatively charged residues were the least favored in docking, with a very low occurrence in ZIKV docked peptides (3% in hexapeptides, 2% in tetrapeptides, and 1% in penta and heptapeptides) and almost no occurrence in DENV peptides (1% in tetra and heptapeptides, 0% in penta and hexapeptides). In general, ZIKV docked peptides displays more positively and negatively charged residues and less aliphatic residues than DENV peptides. A detailed analysis of the amino acid composition of docked ligand can be found in Supplementary Materials (Table S2).

The amino acid distribution of docked peptides was similar to the one found in both ZIKV and DENV envelope protein active site. However, in the proteins, the occurrence of positively and negatively charged amino acid was essentially the same, whereas the docked peptides exhibited more positively than negatively charged residues. This indicates a higher likelihood for peptides to interact with negatively charged residues in the protein glycosylation site rather than with positively charged residues.

The purpose of the virtual screening was the selection of peptides for the specific detection of ZIKV using as counterpart DENV. The rigid body approach used for docking allows the simplification of the screening process in order to process large amount of data in reasonable time. However, it is well-known that molecular docking provides to screen out compounds that did not fit in the binding site or that had grossly wrong electrostatic properties, but it has poor ability to distinguish between the two compounds that both bind to the same active site.

Therefore, in order to reinforce the understanding and the matching between virtual and experimental analysis, three parameters were chosen to select the peptide ligands for experimental evaluation: (1) the position in the top ranked peptides discriminating ZIKV and DENV, (2) the peptide length, and (3) the primary structural analysis of the 0.1% top-ranked peptide ligands.

Table 2a shows the relative docking score position in the corresponding libraries of the eight peptides chosen for the experimental evaluation. The peptide ligands were selected in order to maximize the differences between ZIKV and DENV by considering not the absolute “best” binding scores, but the minimum cross-reactivity between ZIKV and DENV. For all peptides, the differences in ranking scores between the ZIKV and the DENV glycosylation binding sites were large enough to expect a ZIKV selective binding. Nevertheless, only three peptides ranked in the first 10 best peptides in binding ZIKV, highlighting similarities between both glycosylation sites. All the eight peptides ranked outside the top 5% when binding the DENV active site.

We also report in Table 2b the physicochemical properties of the eight peptides selected for the experimental evaluation, with an additional labeling cysteine at the N-terminus of each peptide.

The experimental analysis was performed in PBS at pH 7.4. Therefore, the physicochemical properties were focused on water solubility and net charge at pH 7. Only two peptides had poor water solubility because of the ratio of the hydrophobic amino acids, but when they were used at micromolar concentration both were able to be dissolved in PBS. Five peptides had a significant amount of positively charged amino acids resulting in a positive net charge at pH 7. Because of the presence of the polar amino acids, only two peptides had a slightly negative net charge at pH 7.

Moreover, to highlight the positive or negative charges inside the peptides, the pH of the isoelectric point of each peptide is also reported. Most peptides selected had positively charged amino acids, which are expected to interact with negatively charged residues within the ZIKV glycosylation binding pocket.

As reported previously, the peptide length played a key role in the binding data (Figure 3c). The results suggested the selection of candidates from each of the four libraries for experimental analysis and validation. In fact, a decrease in the docking score was observed from pentapeptides to hexapeptides and from hexapeptides to heptapeptides, owing to steric clashes in the ZIKV and DENV binding cavity. Therefore, two peptide ligands for each library were selected, for a total of eight peptides.

The third parameter considered for the peptide selection was the primary structural analysis. The primary structural analysis was carried out to study the occurrence of the amino acids in the eight selected peptides compared to the top 0.1% ranked peptide ligands in the ZIKV and DENV active sites (160 peptides for the tetrapeptides library, 100 for the pentapeptide, 120 for the hexapeptide and 140 for the heptapeptide, Table S2, in Supplementary Materials). The best 0.1% included only those peptide ligands in the best 1K peptides binding ZIKV that were outside the 5% top peptide ligands binding DENV.

The residue occurrence was calculated counting the recurrence of each amino acid in every relative position in the top ranked peptides (four positions for tetrapeptides, five positions for pentapeptides and so on). Best occurring amino acids were defined as the residue that had the most recurrence for a given position among all peptides in a library. However, peptides having all best occurring amino acids were not present or were in the bottom of the 0.1% top rank peptides.

Table 3 reports the results of the amino acid occurrence (%) in the primary structure of the eight peptides selected for experimental evaluation.

The primary structure in four of the eight selected peptides (LWGH, SWPGQ, KRNATP, and SHRNATA) had an amino acid composition similar to the best occurrence in ZIKV top docked peptides, (21% vs. 23% average amino acid occurrence for LWGH, 24% vs. 27% for SWPGQ, 31% vs. 33% for KRNATP, and 28% vs. 36% for SHRNATA). In the case of the DENV binding site, the amino acid incidence in the four peptides was lower compared with the best occurrence amino acids (10% vs. 26% for LWGH, 8% vs. 35% for SWPGQ, 9% vs. 25% for KRNATP, and 10% vs. 29% for SHRNATA).

The other four selected peptides (QMSK, LRGHA, KTDAYS, GSKANNG) had lower amino acid incidence compared to the average of best occurring amino acids in ZIKV (13% vs. 23% for QMSK, 13% vs. 27% for LRGHA, 14% vs. 33% for KTDAYS, and 11% vs. 36% for GSKANNG).

**Figure 5:**
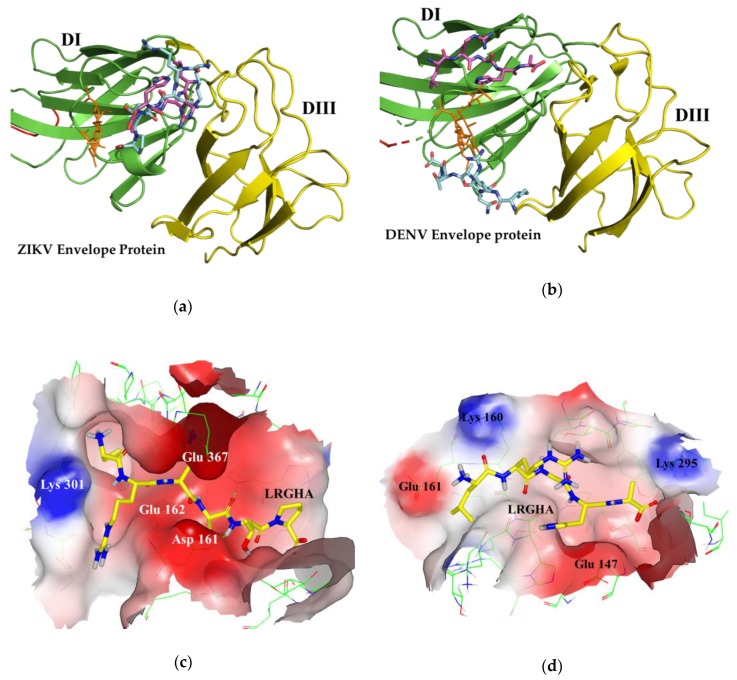

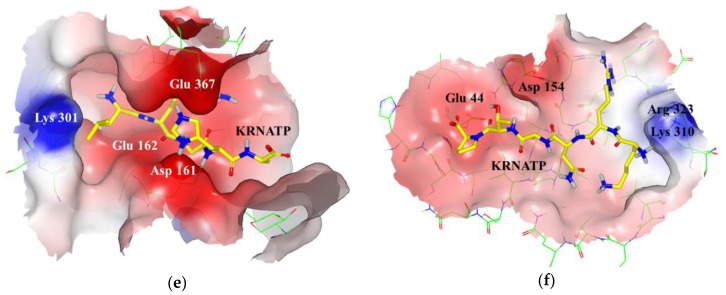
**(a)** ZIKV envelope protein with peptides LRGHA (magenta) and KRNATP (Cyan) docked in the glycosylation binding site. (**b**) DENV envelope protein with peptides LRGHA (magenta) and KRNATP (Cyan) docked in the glycosylation binding site. Protein domains DI, DII, and DIII are depicted in green, red, and yellow, respectively. The glycosylation site residues are highlighted in orange. (**c**) Detailed view of the peptide LRGHA docked to ZIKV binding site. (**d**) Detailed view of the peptide LRGHA docked to DENV binding site. (**e**) Detailed view of the peptide KRNATP docked to ZIKV binding site. (**f**) Detailed view of the peptide KRNATP docked to DENV binding site. The protein surface colors denote the electrostatic charge of binding site residues (white: neutral residues, blue: positively charged residues, red: negatively charged residues).

Nevertheless, these four peptides were chosen for experimental evaluation because they had positions with zero occurrence in DENV top docked peptides (Asn 1 and Lys 4 in QMSK, Arg 2 in LRGHA, Asp 3 and Tyr 5 in KTDAYS, and Arg 3 in SHRNATA). A visual inspection of the three-dimensional peptide–protein complexes revealed differences in the peptides binding ZIKV or DENV (Figure 5). Most peptides docked ZIKV within the 1.6 nm long cavity, with a saddle shaped interaction. This cavity has three negatively charged residues (Asp 161, Glu 162, and Glu 367) and only one positively charged residue (Lys 301) (Figure 5c,e). In comparison, the pocket in DENV is smaller (1.3 nm long) and neutrally charged, possessing two negatively charged residues (Glu 161 and Glu 147) and two positively charged residues (Lys 160 and Lys 295) (Figure 5d,f). These findings explained the differences in affinity of the eight selected peptides to ZIKV and DENV binding site. In fact, among those ligand peptides only LRGHA binds within both ZIKV and DENV pockets (Figure 5c,d). All other ligand peptides bind within the pocket in ZIKV, while in DENV they interact with a planar surface, as observed in Figure 5e,f.

### 3.2. Experimental Results

The eight selected peptides and the reference antibody 4G2, were tested vs. intact ZIKV particles by using a direct ELISA.

All analytical parameters involved in the development of ELISA were optimized by using 96-well plates coated with triplicate 10-fold serial dilutions of intact ZIKV particles. The main results are summarized in Table 4.

To minimize nonspecific binding, four blocking agents (PF, BT, SuperBlock^TM^ blocking buffer, and Blocker™ BSA) were tested. All blocking buffers had very low background signal. For all peptides, except for T2, the lowest background signal was achieved using PF that gave the best performances also using the antibody 4G2. Only for peptide T2, the blocker BT showed better performance probably because that peptide had poor water solubility and zero charge at pH 7. Except for the antibody, no surfactant agent was necessary in the peptide incubation step. No longer than one hour was necessary for peptide incubation, a longer time increased both the overall signal generated by the binding event and the background signal. Shacking during incubation improved the signal to noise ratio.

The optimal concentration of peptide was determined by coating clear 96-well plates with a solution of 10^7^ copies/mL of intact ZIKV particles. Concentrations of peptide, from 0.1 to 50 μM, diluted in 10 mM PBS pH 7.4 were added to wells of the microplates coated with intact ZIKV particles. Larger concentrations than 20 μM did not increase the assay sensitivity. Thus, a peptide concentration of 20 μM was used to estimate the linear range and the limit of detection (LOD) of the assay by using 10-fold serial dilutions of intact ZIKV particles from 10^1^ to 10^9^ copies/mL.

The results had a sigmoidal ZIKV particles concentration response and the calibration curves were obtained by plotting the delta absorbance (after blank signal subtraction) vs. the log of ZIKV particles concentration and fitting the experimental data with a four-parameters logistic function (FPLR).

**Figure 6:**
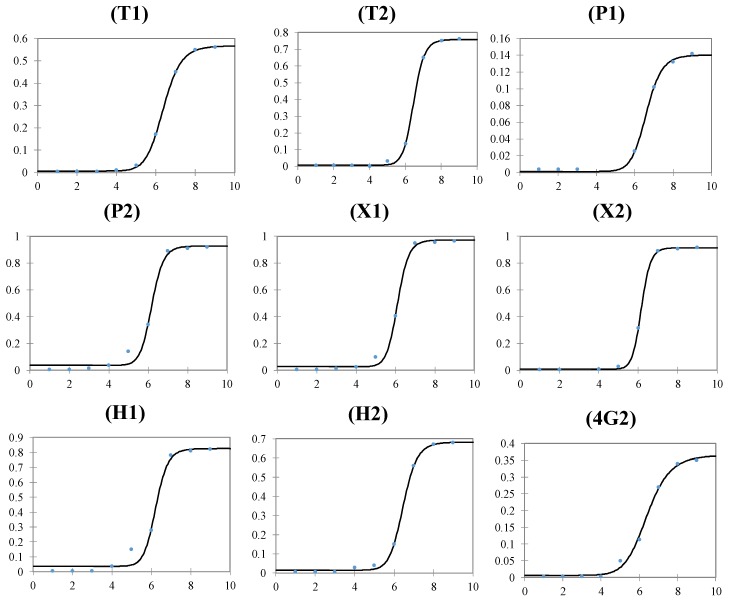
Sigmoidal ZIKV particles concentration response trend of the ELISA assay obtained using the eight peptides and antibody 4G2. *Y*-axis = absorbance (450nm); *X*-axis = log [ZIKV], copies/mL.

The regression parameters of the assay are reported in Table 4 and the sigmoidal trend in Figure 6. The LOD was interpolated from the calibration curves using LOD = Av_B_ + 3 × SD_B_, where Av_B_ and SD_B_ were the average (Av) and the standard deviation (SD) of the blank measurements, respectively.

Dose-response curves generated with all peptides and the antibody had a two-order of magnitude dynamic range except for peptide P1, which had just a one order of magnitude dynamic range. The peptide-based assay using P2, X1, and H1 showed lower detection limits with linear range starting from 10^5^ copies/mL one order magnitude lower than the other peptides or antibody-based assay. The better performance in binding ZIKV intact particles by those three peptides was also highlighted by the FPLR C50 parameter. The dose-response performance of the assay was reproducible over a month (RSD lower than 15%), demonstrating that the peptides had high stability and reproducibility.

The peptides P2, X1, and H1 had similar sensitivity with less amino acids than the decapeptide used in the work of Do Thi Hoang Kim et al. (2018), which had 10^4^ tissue culture infective dose (TCID)_50_/mL (~10^6^ RNA copy number mL^−1^) as limit of detection using a sandwich fluorescence-linked sandwich immunosorbent assay.

In order to increase the performance of the assay, the peptides were bound to the plate by using streptavidin coated plates (Thermo Fisher product 15124) in a sandwich-type assay. Unexpectedly, such scheme produced high background (data not shown), therefore direct ELISA was preferred for further experiments. It should be noted that this work aimed to compare the performances of virtually designed peptides with the biological counterpart and direct ELISA was the easiest protocol to demonstrate the feasibility to work with such synthetic peptides. A comparison between the different bioassays is beyond the scope of this study.

The experimental validation of the selected peptides was addressed to prove that a computational approach can be used to reduce the experimental work for the selection of small peptides for ZIKV detection. The peptide length played an important role in binding the ZIKV particles. Both tetrapeptides gave similar performances, on the contrary the two candidates selected within the penta, hexa, and heptapeptide libraries showed differences in binding ZIKV. The ELISA results were in accordance with the virtual docking score ranking for hexa and heptapeptides but not for pentapeptides. Pentapeptide P1, fourth in virtual binding ZIKV, had the lowest analytical performances, with two orders of magnitude less than the peptide P2 ranked fifty-third in virtual binding ZIKV.

The high occurrence percentage in the primary structure of the top virtual ranked peptides was relevant only in hexapeptides. Hexapeptide X1, with an amino acid composition similar to the best occurrence in ZIKV top docked peptides, was one of the three peptides with the best ELISA assay performances.

The cross-reactivity among Flaviviruses is a key parameter to be tested for this assay. Using the same ELISA protocol, the three peptides with higher sensitivity versus the intact ZIKV particles where employed to test the ability to discriminate ZIKV from the three serotypes of DENV (DENV-1, -2, and -3).

**Figure 7:**
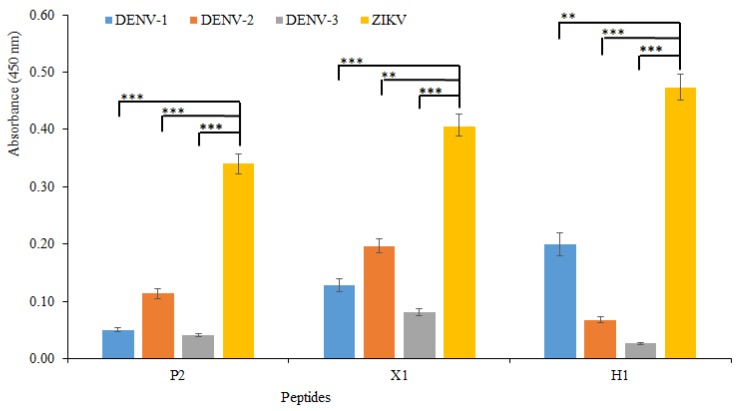
Cross-reactivity study. In the ELISA direct assay, the spectrophotometric absorbance signals were obtained by using the best three peptides (P2, X1, and H1) binding the ZIKV and three serotypes of DENV (DENV-1, -2, and -3) at the concentration of 10^6^ copies/mL. Statistical significance between ZIKV and DENV serotypes (1−3) was calculated using two-way analysis of variance. Different p values were indicated by **(*p* < 10^−3^) or ***(*p* < 10^−4^).

The results shown in Figure 7 were obtained by coating clear 96-well plates with a solution of 10^6^ copies/mL of intact virus particles. At this concentration, all three peptides showed slight cross-reactivity against the DENV. DENV-2 had the higher DENV/ZIKV signal ratio for peptides P2 and X1 34% and 49%, respectively, while peptide X1 cross-reacted with DENV-1 with a 32% of DENV/ZIKV signal ratio.

Peptide H1 showed significant cross-reactivity only with DENV-1 with a DENV/ZIKV signal ratio up to 42%. Nevertheless, the three peptides clearly discriminated between the two flavivirus species. In all the three peptides, reactivity against ZIKV was significantly different from cross-reactivity against DENV at 10^6^ copies/mL of intact virus particles, with p-values ranging from <10^−3^ to <10^−4^. It is important to note that during Zika infection, the concentration of ZIKV in serum, saliva and urine can rise to around 10^6^ copies/mL [44,45]. Therefore, the peptides could be suitable for discriminating between ZIKV and DENV at physiological levels.

Analytical signals were statistically equivalent, increasing the coating concentration of the virus to 10^7^ copies/mL, losing the discrimination between the DENV and ZIKV. In contrast, the peptide used in the work of Do Thi Hoang Kim et al. (2018) displayed ZIKV selectivity at concentrations of 2.25 × 10^6^ RNA copies/mL and maintained this selectivity when the virus concentration was increased ten-fold.

Usually, the presence of ZIKV in affected bodies is detected in biological fluids. Therefore, the analytical sensitivity of the selected peptides was tested in two biological matrices, namely, urine and serum. The matrix effect was investigated to understand how real biological fluids could modify the binding efficiency of the peptides.

**Figure 8:**
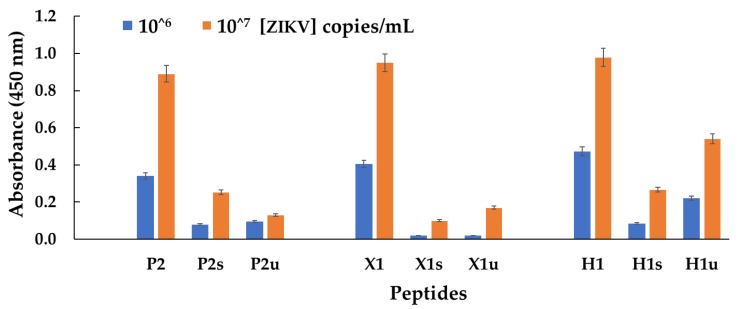
Urine (u) and serum (s) matrix effect on the ELISA signal using the best three peptides (P2, X1, and H1) binding ZIKV at the concentrations of 10^6^ and 10^7^ copies/mL.

Figure 8 reports the ELISA data using solutions of peptides with or without the urine and serum obtained, coating clear 96-well plates with 10^6^ and 10^7^ copies/mL of intact ZIKV particles. Serum and urine were 1:10 and 1:1 diluted, respectively, with a concentrated peptide PBS solution to obtain a peptide final concentration of 20 μM (10 mM PBS, pH 7.4). A coating concentration of 10^5^ was also tested, but no signal was observed after the addition of the biological matrix.

Peptide P2 showed a reduction in absorbance in the presence of the biological matrices, with an observed signal decrease of 77% for serum and 72% for urine at 10^6^ copies/mL of intact ZIKV particles. At 10^7^ copies/mL, P2 showed better performance in serum (signal decrease of 72%) than urine (signal decrease of 85%). Peptide X1 had a strong reduction in the signal generated at 10^6^ copies/mL in both urine and serum with an observed signal decrease of 95% for both matrices. The detection limit of this peptide was 10^7^ copies/mL. At this concentration, the performance was better in urine (signal decrease of 82%) than in serum (signal decrease of 89%). Peptide H1 exhibited the best performance among the three peptides, having higher signals in urine (signal decrease of 53% at 10^6^ copies/mL, 45% at 10^7^ copies/mL) than in serum (signal decrease of 82% at 10^6^ copies/mL, 73% at 10^7^ copies/mL). The sensitivity of the three peptides decreased at the least one order of magnitude when detecting ZIKV in urine or serum. It should be highlighted that during the viremia peak of Zika infection, the lowest ZIKV concentration is 10^6.9^ copies/mL in serum, close to the limit of detection showed by peptides P2 and H1 [44,45].

## 4. Conclusions

The semi combinatorial virtual strategy to design peptides using the flavivirus glycosylation site as a binding target has shown to have the potential for designing candidates for the selective detection of ZIKV.

The direct ELISA platform developed for testing the newly designed peptides offered the possibility to optimize in short time the experimental conditions for evaluation of the eight peptides chosen from the most promising ones yield by the in silico studies.

The three parameters chosen to select the peptide ligands for experimental evaluation, helped to understand the matching between in silico selection and experimental results.

The virtual docking score ranking was in accordance with the experimental evidence only for hexa and heptapeptides highlighting the importance of the peptide length. In fact, the two candidates selected within the penta, hexa, and heptapeptide libraries showed differences in binding ZIKV. The high occurrence percentage in the primary structure of the top virtual ranked peptides was relevant only in hexapeptides, further investigations are needed to confirm the role of this parameter.

This work confirmed that the performances of virtually designed peptides were similar or even better than the biological counterpart, the antibody 4G2.

The three peptides with best performances in ZIKV detection also showed semi-selective properties when tested against DENV. However, the reactivity against ZIKV was statistically different from cross-reactivity against DENV only when the coating concentration of the virus was less than 10^7^ copies/mL

The matrix-effect was also investigated, by testing the response of the peptides in physiological matrices, serum and urine. We observed that the matrix affected the assay performance by decreasing the detection limits by one order of magnitude, albeit still having for two of the three peptides tested a distinct analytical signal starting from 10^6^ copies/mL, the concentration of ZIKV in acute infection.

This work represents a new methodology for the selection of tailor-made peptides, rationalizing the way to choose receptors with high binding ability among thousands of potential compounds that can be employed in biotechnology and analytical applications. Taking advantage of the fast progress in computing, we envision that it will be possible to simulate peptides having more complex shapes with better selectivity and less cross-reactivity.

In our opinion, the computationally assisted screening of molecular structures could narrow to a general method to obtain tailor-made reagents, avoiding very large procedures like combinatorial work or trial and error method.

In conclusion, these results demonstrate the proof-of-concept of working with rationally designed peptides and support the field of virtually assisted bioanalysis.

## Figures and Tables

**Table 1:** (**a**) Occurrence analysis (%) in ZIKV and DENV envelope protein binding site residues (defined by the outer docking contour) grouped by using side chain type. (**b**) Occurrence analysis (%) in the primary sequence of the top 0.1% ranked peptide ligands grouped by using side chain type. The numbers represent the average percentage of amino acid positions belonging to each one of the different side chain type.

**(a)**	**Occurrence (%)**			
	**ZIKV**	**DENV**		
**Side chain type**	**Receptor Active Site**			
Aliphatic	45	49		
Polar	26	19		
Aromatic	4	4		
Negative	12	13		
Positive	13	15		
**(b)**	**Occurrence (%)**		**Occurrence (%)**	
	**ZIKV**	**DENV**	**ZIKV**	**DENV**
**Side chain type**	**Tetrapeptide**		**Pentapeptide**	
Aliphatic	44	61	51	60
Polar	31	19	32	32
Aromatic	8	5	7	2
Negative	2	1	1	0
Positive	14	14	9	6
	**Hexapeptide**		**Heptapeptide**	
Aliphatic	50	46	49	60
Polar	33	42	36	32
Aromatic	5	8	4	4
Negative	3	0	1	1
Positive	10	3	9	3

**Table 2:** (**a**) Relative docking score position in the corresponding libraries of the eight peptides selected for experimental evaluation versus ZIKV and DENV envelope protein glycosylation binding site. (**b**) Physicochemical properties of the eight peptides selected for experimental part. A cysteine residue was added to the N terminus of each peptide to link the maleimide-PEG2-biotin. Iso-Point = isoelectric point. Water Sol. = water solubility. MW = molecular weight.

(a)	Docking Score Rank		(b)					
Peptide in Simulation	ZIKV	DENV	Peptide in Experimental	Label	Iso-Point (pH)	Net Charge at pH 7	Water Sol.	MW
**QMSK**	15	32607	**C-QMSK**	T1	9.13	0.9	good	595
**LWGH**	48	101726	**C-LWGH**	T2	7.09	0.0	poor	614
**SWPGQ**	4	55575	**C-SWPGQ**	P1	2.98	−0.1	poor	676
**LRGHA**	53	74900	**C-LRGHA**	P2	9.21	1.0	good	655
**KRNATP**	16	85123	**C-KRNATP**	X1	10.46	1.9	good	788
**KTDAYS**	120	95558	**C-KTDAYS**	X2	5.92	−0.1	good	786
**GSKANNG**	1	63937	**C-GSKANNG**	H1	9.13	0.9	good	749
**SHRNATA**	5	94782	**C-SHRNATA**	H2	9.21	1.0	good	858

**Table 3:** Analysis of the amino acid occurrence (%) in the primary structure of the eight peptides selected for experimental evaluation. The occurrence was calculated counting the recurrence of each amino acid in the relative position (four positions for tetrapeptides, five positions for pentapeptides, and so on) in the top 0.1% ranked peptides binding ZIKV and DENV envelope protein. The best occurring amino acids were also reported (in italic) along with the average (Av) percentage of the occurrence for each peptide.

ZIKV		**1P**	**2P**	**3P**	**4P**	**Av**			
	**QMSK**	21	10	8	11	13			
	**LWGH**	17	11	39	16	21			
	***best occurring AA: QSGH***	*21*	*18*	*39*	*16*	*23*			
DENV		**1P**	**2P**	**3P**	**4P**	**Av**			
	**QMSK**	0	3	4	0	2			
	**LWGH**	1	0	36	1	10			
	***best occurring AA: GPGP***	*26*	*21*	*36*	*21*	*26*			
ZIKV		**1P**	**2P**	**3P**	**4P**	**5P**	**Av**		
	**SWPGQ**	29	18	31	39	2	24		
	**LRGHA**	11	6	21	21	8	13		
	***best occurring AA: SWPGG***	*29*	*18*	*31*	*39*	*19*	*27*		
DENV		**1P**	**2P**	**3P**	**4P**	**5P**	**Av**		
	**SWPGQ**	8	1	10	20	0	8		
	**LRGHA**	21	0	13	7	12	11		
	***best occurring AA: LGASG***	*21*	*38*	*35*	*43*	*38*	*35*		
ZIKV		**1P**	**2P**	**3P**	**4P**	**5P**	**6P**	**Av**	
	**KRNATP**	10	6	32	57	37	43	31	
	**KTDAYS**	10	10	3	57	3	3	14	
	***best occurring AA: FPNATP***	13	14	32	57	37	43	33	
DENV		**1P**	**2P**	**3P**	**4P**	**5P**	**6P**	**Av**	
	**KRNATP**	2	0	2	34	18	2	9	
	**KTDAYS**	2	2	0	34	0	12	8	
	***best occurring AA: GSSASC***	*18*	*27*	*33*	*34*	*21*	*19*	*25*	
ZIKV		**1P**	**2P**	**3P**	**4P**	**5P**	**6P**	**7P**	**Av**
	**GSKANNG**	27	3	6	14	7	6	11	11
	**SHRNATA**	9	13	12	36	66	50	14	28
	***best occurring AA: GFPNATP***	*27*	*19*	*16*	*36*	*66*	*50*	*40*	*36*
DENV		**1P**	**2P**	**3P**	**4P**	**5P**	**6P**	**7P**	**Av**
	**GSKANNG**	40	8	0	9	6	1	14	11
	**SHRNATA**	13	1	0	11	19	24	5	10
	***best occurring AA: GGPTPGP***	*40*	*36*	*25*	*19*	*22*	*27*	*36*	*29*

**Table 4:** Optimized experimental parameters of the direct enzyme linked immunosorbent assay (ELISA) assay obtained using the eight peptides and the commercial antibody (4G2). BT = BLOTTO blocking buffer; PF = protein free blocking buffer; PBS = 10 mM phosphate buffer saline pH 7.4; PBST = 10 mM PBS pH 7.4, 0.1% Tween-20. FLRP = four parameters logistic regression; CV = coefficient of variation.

		T1	T2	P1	P2	X1	X2	H1	H2	4G2
**Blocking**		PF	BT	PF	PF	PF	PF	PF	PF	PF
**Incubation buffer**		PBS	PBS	PBS	PBS	PBS	PBS	PBS	PBS	PBST
**FPLR—Dynamic Range**	**(log[ZIKV], copies/mL)**	6-8	6-8	7-8	5-7	5-7	6-8	5-7	6-8	6-8
**LOD**	**(log[ZIKV], copies/mL)**	5.8	5.8	6.8	4.7	4.8	5.8	4.5	5.7	5.8
**FPLR—C50**	**(log[ZIKV], copies/mL)**	6.4	6.5	6.6	6.1	6.1	6.2	6.2	6.5	6.4
**FPLR—slope**	**Absorbance/(log[ZIKV], copies/mL)**	14.2	21.7	16.3	20.9	21.9	26.7	20.4	18.3	10.7
**FPLR—maximum**	**Absorbance (450 nm)**	0.568	0.759	0.140	0.928	0.973	0.915	0.826	0.683	0.365
**FPLR—minimum**	**Absorbance (450 nm)**	0.007	0.008	0.001	0.036	0.026	0.007	0.036	0.014	0.007
**FPLR- R^2^**		1.000	0.999	0.997	0.991	0.996	1.000	0.987	0.999	0.996
**Peptide Concentration**	**(μM)**	20	20	20	20	20	20	20	20	1 μg/mL
**Intra-day reproducibility**	**CV(%)**	<5	<5	<5	<5	<5	<5	<5	<5	<10
**Inter-day and batch-to-batch reproducibility**	**CV(%)**	<10	<10	<10	<10	<10	<10	<10	<10	nd
**Long-term stability**	**(Month)**	>1	>1	>1	>1	>1	>1	>1	>1	nd
**Assay time after Plate Coating**	**(h)**	5	5	5	5	5	5	5	5	8

## Supplementary Materials

The following are available online at https://www.mdpi.com/2218-273X/9/9/498/s1, Table S1: Amino acids residues surrounding the ZIKV and DENV glycosylation site. Table S2: Primary structural analysis of the top 0.1% ranked peptides.
