# Supplementary material for: Computationally Designed Peptides for Zika Virus Detection: An Incremental Construction Approach
Source: Biomolecules. 2019 Sep 17;9(9):498. doi: 10.3390/biom9090498 (PMC6770336; doi:10.3390/biom9090498)
Supplement: Supplementary file 1 [file biomolecules-09-00498-s001.pdf]

Table S1. The amino acid (AA) residues surrounding the ZIKV and DENV glycosylation site (defined by the outer docking contour) used for statistical analysis to highlight the differences in amino acid composition between both binding sites. The residues sequence number in the ZIKV and DENV envelope proteins was also reported. The residues were grouped according to their side chain type.

		ZIKV		DENV	
**Side chain type**	**AA**	**AA Count**	**AA Sequence number**	**AA Count**	**AA Sequence number**
Aliphatic	**ALA**	5	35, 165, 178, 319, 361	3	150, 313, 369
	**GLY**	9	5, 28, 29, 145, 150, 157, 181, 182, 184	9	5, 28, 146, 152, 156, 159, 177, 296, 318
	**ILE**	8	1, 4, 43, 139, 152, 317, 359, 365	15	4, 6, 23, 46, 139, 141, 162, 308, 312, 320, 322, 335, 352, 357, 367
	**LEU**	9	25, 45, 141, 180, 186, 298, 300, 376, 378	10	25, 41, 45, 175, 277, 278, 283, 287, 292, 294
	**MET**	5	34, 140, 151, 374, 375	3	1, 34, 289
	**PRO**	4	39, 318, 338, 363	4	39, 143, 356, 364
	**VAL**	13	6, 12, 31, 33, 41, 46, 143, 153, 167, 326, 328, 330, 364	11	12, 21, 24, 31, 140, 151, 309, 321, 324, 354, 365
Polar	**CYS**	2	3, 30	2	3, 30
	**ASN**	3	8, 154, 163	3	8, 153, 366
	**GLN**	2	147, 331	2	43
	**SER**	8	7, 142, 146, 149, 185, 285, 368, 372	3	7, 29, 145
	**THR**	15	32, 40, 47, 48, 156, 160, 179, 313, 315, 321, 325, 327, 360, 366, 369	11	32, 33, 40, 48, 138, 142, 155, 176, 315, 319, 353
Aromatic	**PHE**	3	11, 183, 314	2	11, 43
	**TRP**	0		1	391
	**TYR**	2	137, 332	2	137, 178
Negatively Charged	**ASP**	5	10, 42, 155, 161, 379	4	10, 22, 42, 154
	**GLU**	9	26, 44, 159, 162, 168, 177, 329, 367, 377	10	26, 44, 49, 136, 147, 148, 161, 311, 314, 368
Positively Charged	**HIS**	4	27, 144, 148, 158	5	27, 144, 149, 158, 317
	**LYS**	6	38, 166, 297, 301, 316, 373	9	38, 47, 157, 160, 163, 284, 295, 307, 310
	**ARG**	5	2, 9, 138, 164, 299	3	2, 9, 323

Table S2. The primary structural analysis carried out to study the occurrence of the amino acids in the top 0.1% ranked peptides. The chemical class aminoacid occurrence was reported and highlighted in different colors. The colorimetric scale was added to the table, to highlight differences in aminoacid occurrence. The aminoacids within the eight peptides selected for the experimental part were evaluated comparing the occurrence to the one of best statistical aminoacid in the corresponding position (best stat. AA).

	ZIKV						DENV				
AA	1P	2P	3P	4P			1P	2P	3P	4P	
ALA	5	8	32	8			22	21	12	24	
CYS	4	12	3	7			4	10	13	7	
ASP	1	2	2	2			4	0	0	0	
GLU	0	1	4	0			2	0	1	0	
PHE	6	2	0	2			4	7	0	1	
GLY	0	10	63	11			42	28	58	22	
HIS	10	9	9	25			3	1	1	1	
ILE	4	4	0	8			2	1	2	7	
LYS	1	1	0	18			8	16	18	0	
LEU	27	1	0	4			2	2	7	11	
MET	0	16	1	0			0	5	6	1	
ASN	8	1	3	12			5	2	5	0	
PRO	6	23	12	19			5	33	17	34	
GLN	33	0	0	2			0	0	0	0	
ARG	7	9	0	0			17	10	1	13	
SER	10	29	13	25			10	5	7	10	
THR	10	7	15	7			17	5	3	18	
VAL	9	6	1	6			2	8	8	9	
TRP	12	18	0	0			1	0	0	0	
TYR	7	1	2	4			10	6	1	2	
	**160**	**160**	**160**	**160**	**Av**		**160**	**160**	**160**	**160**	**Av**
Aliphatic	32%	43%	68%	35%	**44%**	Aliphatic	47%	61%	69%	68%	**61%**
Polar	41%	31%	21%	33%	**31%**	Polar	23%	14%	18%	22%	**19%**
Aromatic	16%	13%	1%	4%	**8%**	Aromatic	9%	8%	1%	2%	**5%**
Negative	1%	2%	4%	1%	**2%**	Negative	4%	0%	1%	0%	**1%**
Positive	11%	12%	6%	27%	**14%**	Positive	18%	17%	13%	9%	**14%**
	**QMSK**	**Q**	**M**	**S**	**K**		**QMSK**	**Q**	**M**	**S**	**K**
		21%	10%	8%	11%			0%	3%	4%	0%

Peptides selected for experimental	LWGH	L	W	G	H
		17%	11%	39%	16%
	best stat AA	Q	S	G	H
		21%	18%	39%	16%

ZIKV

AA	1P	2P	3P	4P	5P	
ALA	12	8	27	3	8	
CYS	2	3	2	3	11	
ASP	0	0	0	0	1	
GLU	0	0	0	1	1	
PHE	1	9	0	0	0	
GLY	6	1	21	39	19	
HIS	1	4	0	21	3	
ILE	4	2	0	0	3	
LYS	1	2	0	0	0	
LEU	11	1	0	1	5	
MET	0	14	0	0	2	
ASN	6	2	5	2	5	
PRO	2	0	31	1	9	
GLN	13	1	0	0	2	
ARG	3	6	2	3	0	
SER	29	13	7	4	10	
THR	0	7	5	22	8	
VAL	7	4	0	0	13	
TRP	1	18	0	0	0	
TYR	1	5	0	0	0	
	100	100	100	100	100	Av
Aliphatic	42%	30%	79%	44%	59%	51%
Polar	50%	26%	19%	31%	36%	32%
Aromatic	3%	32%	0%	0%	0%	7%
Negative	0%	0%	0%	1%	2%	1%
Positive	5%	12%	2%	24%	3%	9%
	SWPGQ	S	W	P	G	Q

	LWGH	L	W	G	H
		1%	0%	36%	1%
	best stat AA	G	P	G	P
		26%	21%	36%	21%

DENV

AA	1P	2P	3P	4P	5P	
ALA	6	7	35	11	12	
CYS	8	8	4	1	4	
ASP	1	0	1	0	0	
GLU	0	0	0	0	0	
PHE	3	0	0	0	2	
GLY	8	38	13	20	38	
HIS	0	1	0	7	4	
ILE	3	4	2	0	1	
LYS	2	1	3	10	0	
LEU	21	2	1	0	0	
MET	0	0	1	0	0	
ASN	7	1	1	0	8	
PRO	8	16	10	8	9	
GLN	1	1	0	0	0	
ARG	2	0	0	0	0	
SER	8	15	17	43	12	
THR	8	2	7	0	4	
VAL	12	2	5	0	5	
TRP	1	1	0	0	0	
TYR	1	1	0	0	1	
	100	100	100	100	100	Av
Aliphatic	58%	69%	67%	39%	65%	60%
Polar	32%	27%	29%	44%	28%	32%
Aromatic	5%	2%	0%	0%	3%	2%
Negative	1%	0%	1%	0%	0%	0%
Positive	4%	2%	3%	17%	4%	6%
	SWPGQ	S	W	P	G	Q

		ZIKV						DENV				
**Peptides selected for experimental**		29%	18%	31%	39%	2%		8%	1%	10%	20%	0%
	**LRGHA**	L	R	G	H	A	**LRGHA**	L	R	G	H	A
		11%	6%	21%	21%	8%		21%	0%	13%	7%	12%
	**best stat AA**	S	W	P	G	G	**best stat AA**	L	G	A	S	G
		29%	18%	31%	39%	19%		21%	38%	35%	43%	38%

AA	ZIKV 1P	ZIKV 2P	ZIKV 3P	ZIKV 4P	ZIKV 5P	ZIKV 6P	DENV 1P	DENV 2P	DENV 3P	DENV 4P	DENV 5P	DENV 6P
ALA	14	8	7	68	4	7	11	15	20	41	22	21
CYS	9	9	0	1	3	6	3	2	11	4	7	23
ASP	1	0	3	4	1	2	0	0	0	0	1	1
GLU	0	7	0	0	0	0	0	0	0	1	0	0
PHE	15	1	0	0	0	2	2	5	0	0	0	10
GLY	14	5	13	8	19	12	21	9	3	8	19	21
HIS	7	2	0	2	4	7	2	2	0	0	2	0
ILE	7	4	1	2	3	2	7	2	1	0	1	0
LYS	12	1	9	0	0	0	2	0	1	0	11	0
LEU	6	9	2	0	0	0	11	4	0	0	0	0
MET	3	3	1	0	0	5	13	2	0	0	0	2
ASN	1	7	38	5	3	10	6	1	2	2	1	4
PRO	11	17	8	11	6	52	2	7	22	9	2	2
GLN	0	5	1	0	1	0	4	1	0	2	0	0
ARG	4	7	11	1	2	0	3	0	0	2	0	0
SER	10	7	6	3	21	3	21	32	39	34	25	14
THR	6	12	15	11	44	0	4	2	21	14	21	4
VAL	0	11	4	1	3	6	8	5	0	2	8	9
TRP	0	5	0	0	3	0	0	17	0	1	0	0
TYR	0	0	1	3	3	6	0	14	0	0	0	9

ZIKV

	120	120	120	120	120	120	Av
Aliphatic	46%	48%	30%	75%	29%	70%	50%
Polar	22%	33%	50%	17%	60%	16%	33%
Aromatic	13%	5%	1%	3%	5%	7%	5%
Negative	1%	6%	3%	3%	1%	2%	3%
Positive	19%	8%	17%	3%	5%	6%	10%
	KRNATP	K	R	N	A	T	P

DENV

	120	120	120	120	120	120	Av
Aliphatic	61%	37%	38%	50%	43%	46%	46%
Polar	32%	32%	61%	47%	45%	38%	42%
Aromatic	2%	30%	0%	1%	0%	16%	8%
Negative	0%	0%	0%	1%	1%	1%	0%
Positive	6%	2%	1%	2%	11%	0%	3%
	KRNATP	K	R	N	A	T	P

Peptides selected for experimental		10%	6%	32%	57%	37%	43%		2%	0%	2%	34%	18%	2%
	KTDAYS	K	T	D	A	Y	S	KTDAYS	K	T	D	A	Y	S
		10%	10%	3%	57%	3%	3%		2%	2%	0%	34%	0%	12%
	best stat AA	F	P	N	A	T	P	best stat AA	G	S	S	A	S	C
		13%	14%	32%	57%	37%	43%		18%	27%	33%	34%	21%	19%

ZIKV

AA	1P	2P	3P	4P	5P	6P	7P	
ALA	21	8	2	19	93	2	19	
CYS	17	6	8	2	1	1	19	
ASP	1	0	0	0	1	4	0	
GLU	0	4	3	0	0	1	0	
PHE	2	27	0	1	0	0	0	
GLY	38	9	16	1	6	12	15	
HIS	3	18	1	0	1	10	4	
ILE	2	5	9	0	1	0	3	
LYS	4	0	8	8	1	0	2	
LEU	1	5	18	1	1	3	0	
MET	2	3	3	2	1	0	0	
ASN	1	17	12	50	10	9	7	
PRO	20	14	22	6	9	4	56	
GLN	0	7	8	0	0	1	0	
ARG	3	2	17	1	6	0	0	
SER	12	4	3	3	2	20	3	
THR	11	2	4	39	4	70	2	
VAL	2	4	4	3	3	1	9	
TRP	0	0	2	0	0	0	0	
TYR	0	5	0	4	0	2	1	
	140	140	140	140	140	140	140	Av
Aliphatic	61%	34%	53%	23%	81%	16%	73%	49%
Polar	29%	26%	25%	67%	12%	72%	22%	36%
Aromatic	1%	23%	1%	4%	0%	1%	1%	4%
Negative	1%	3%	2%	0%	1%	4%	0%	1%
Positive	7%	14%	19%	6%	6%	7%	4%	9%
	GSKANNG	G	S	K	A	N	N	G

DENV

AA	1P	2P	3P	4P	5P	6P	7P	
ALA	19	12	24	12	26	9	7	
CYS	10	3	2	15	3	9	8	
ASP	0	0	0	0	4	0	3	
GLU	0	0	0	0	0	0	0	
PHE	1	0	0	0	1	0	12	
GLY	56	51	11	6	16	38	19	
HIS	3	1	0	0	0	3	5	
ILE	1	6	2	14	10	2	0	
LYS	3	1	0	1	0	0	1	
LEU	1	1	11	6	0	0	0	
MET	0	1	1	2	12	3	1	
ASN	7	28	18	15	8	2	4	
PRO	7	7	35	22	31	18	51	
GLN	0	3	0	0	0	2	0	
ARG	0	4	0	1	0	6	0	
SER	18	11	16	10	8	10	2	
THR	13	2	11	26	16	34	1	
VAL	1	9	9	10	3	2	2	
TRP	0	0	0	0	0	0	0	
TYR	0	0	0	0	2	2	24	
	140	140	140	140	140	140	140	Av
Aliphatic	61%	62%	66%	51%	70%	51%	57%	60%
Polar	34%	34%	34%	47%	25%	41%	11%	32%
Aromatic	1%	0%	0%	0%	2%	1%	26%	4%
Negative	0%	0%	0%	0%	3%	0%	2%	1%
Positive	4%	4%	0%	1%	0%	6%	4%	3%
	GSKANNG	G	S	K	A	N	N	G

**Peptides selected for experimental**		27%	3%	6%	14%	7%	6%	11%
	**SHRNATA**	**S**	**H**	**R**	**N**	**A**	**T**	**A**
		9%	13%	12%	36%	66%	50%	14%
	**best stat AA**	**G**	**F**	**P**	**N**	**A**	**T**	**P**
		27%	19%	16%	36%	66%	50%	40%

	40%	8%	0%	9%	6%	1%	14%
**SHRNATA**	**S**	**H**	**R**	**N**	**A**	**T**	**A**
	13%	1%	0%	11%	19%	24%	5%
**best stat AA**	**G**	**G**	**P**	**T**	**P**	**G**	**P**
	40%	36%	25%	19%	22%	27%	36%
